# Growth Factor Applied to Oral and Regenerative Surgery

**DOI:** 10.3390/ijms21207752

**Published:** 2020-10-20

**Authors:** Marco Cicciù

**Affiliations:** Department of Biomedical and Dental Sciences, Morphological and Functional Images, University of Messina, 98100 Messina, Italy; mcicciu@unime.it

**Keywords:** bone, oral surgery, implantology, periodontology, growth factor, regenerative surgery

## Abstract

The complex tissue engineering/regenerative medicine now represents a therapeutic reality applicable to various organic substrates, with the aim of repairing deficient tissues and restoring normal organ function. Among the possible specialized uses, in the dental field, the treatment of periodontal, pre- and peri-implant bone defects should be mentioned. Nowadays, in oral surgery, there are many surgical methods that can be used, despite that the literature still seems controversial regarding the actual advantages of their use. Surely, this work will bring to light the current clinical-surgical orientations and the different perspectives.

In biology, the term growth factor refers to proteins capable of stimulating cell proliferation, cell differentiation and preventing apoptosis [1,2]. They are typical signal molecules used for communication between the cells of an organism; for example, cytokines or hormones that bind to specific receptors on the cell membrane of their targets [3].

The main function of growth factors is the external control of the cell cycle, through the abandonment of cellular quiescence (phase G0) and the entry of the cell into phase G1 (growth) [4,5,6,7]. However, this is not their only function; in fact, they regulate the entry into mitosis, cell survival, cell migration and differentiation. Together with proliferation, they always simultaneously promote differentiation and maturation [8]. The individual growth factors tend to group into large families of structurally and evolutionarily similar proteins. Famous are, for example, the families of insulin-like growth factors (IGF), transforming growth factor, bone morphogenetic protein (BMP) [9], neurotrophins (NGF, BDNF and NT3), FGF (growth factor of fibroblasts) and so on. The first attempts to use growth factors in dentistry date back to the introduction of platelet-rich plasma (PRP), plasma rich in growth factors (PRGF) and plasma rich in fibrin (PRF). However, their effectiveness has not always been clearly documented. More recently, recombinant growth factors have been analyzed and used in dentistry, including recombinant growth factor derived from human platelets (rh PDGF-BB) and bone morphogenetic proteins (BMPs) (Figure 1). In the dental field, bone regeneration, if not for major reconstructions, which fall within the maxillofacial area, is often the basis of occlusal rehabilitation, hence the possibility of using and guaranteeing osseointegration of dental implants. Today, osseointegrated dental implants represent a well-documented, standardized and highly predictable treatment. Significant bone deficits in the areas intended to receive the implant can limit this procedure. For this reason, numerous bone regeneration techniques have been developed such as guided regeneration (GBR), alveolar osteodistraction, the use of titanium meshes and both block and particulate grafts. These procedures can include both the contextual insertion of the implant (one-stage techniques) and the deferred insertion in case of major bone deficits (two-stage techniques). However, in the literature, early and late failures of the regeneration techniques and significant complications related to the morbidity of the donor and recipient site are described. To obviate the aforementioned complications and make regenerative techniques highly predictable, research has focused on the use of autologous growth factors as the best way to induce tissue regeneration:

- Platelet-derived growth factor (PDGF);
- Transforming growth factor-β 1 (TGF-β 1) and β 2 (TGF-β 2);
- Fibroblast growth factor (FGF);
- Vascular endothelial growth factor (VEGF);
- Insulin-like growth factor (IGF).

They stimulate cell proliferation, remodeling of the extra-cellular matrix and angiogenesis.

As previously specified, guided bone regeneration (GBR) maneuvers involve the use of different medical devices to obtain their results. In fact, it is not always sufficient to use only these growth factors. Underlying GBR is the need to selectively “guide” tissue healing. In fact, by exploiting the different growth turnover of the different cytotypes, it is possible to screen the growth of the latter, and promote the “undisturbed” growth of slower-growing tissues [10,11,12,13]. For example, in the periodontal setting, the growth turnover of soft tissues is much faster than that of hard tissue growth and maturation (bone). To make this possible, it is necessary to use semipermeable membranes, which allow the exchange of gas and nutrients between the tissues but prevent cell penetration and proliferation. Membranes can be classified into resorbable and non-resorbable according to surgical needs, and they may or may not be reinforced. However, it is also necessary to have a scaffold that can stabilize the clot and heal the tissues in an undisturbed way, as well as support the membrane. The scaffold at is represented by:

- Autologous bone;
- Homologous bone;
- Heterologous bone;
- Alloplastic materials.

Alloplastic materials include hydroxyapatites, tricalcium beta-phosphate, bio-glasses and marine-derived biomaterials [14,15]. Growth factors are often used in association with the scaffold, in close contact with the surfaces that guide tissue healing, precisely with the aim of improving it. Table 1 is the result of a recent systematic review which shows which compositions are most used and tested to perform bone regeneration according to different techniques [9].

Growth factors certainly influence bone regeneration and an increase in their concentration involves both an acceleration in the formation of bone and an increase in its quantity. In conclusion, it can be said that, for bone tissue, in vivo regeneration techniques are to be preferred, preferably using autologous bone as an osteoconductive and osteogenic matrix considering that the bone structure is also the result of the biomechanical environment in which the bone graft develops and matures. In conclusion, growth factors can improve surgical outcomes (improvement in bone height and thickness) compared to conventional techniques (without the use of growth factors), both in the systemic field of the patient (improvement in quality of life) [3,16,17,18,19,20,21]. Of course, further studies are still needed to analyze in detail the differences between different growth factors and their performance.

## Figures and Tables

**Figure 1:**
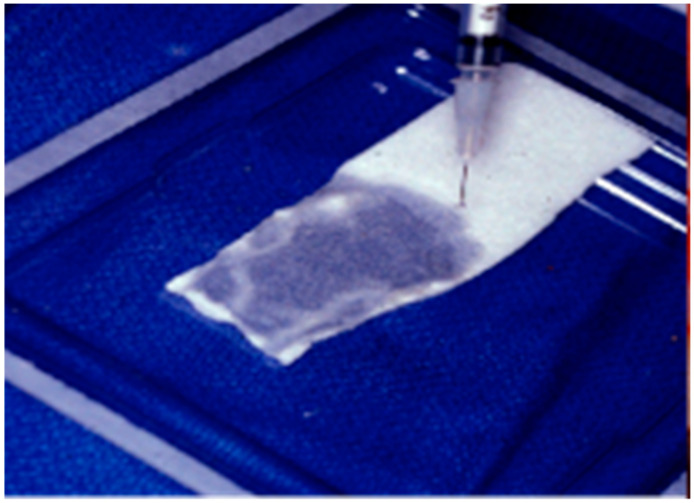
Bone morphogenetic protein (BMP)-loaded collagen membrane.

**Table 1:** Commonly used growth factor compound.

Xenogenic bone block loaded with rhBMP-2
GBR using acellular dermal matrix (ADM) film combined with alveolar bone grafting vs. alveolar bone grafting combined with concentrated growth factor (CG).
Bilateral maxillary sinus and ridge augmentation procedures using rhBMP-2 combined with allograft and xenograft.
Horizontal ridge augmentation surgery with deproteinized bovine bone xenograft particles combined with rhPDGF BB with collagen membrane.
rhPDGF-BB GBR with beta-tricalcium phosphate (β-TCP)/hydroxyapatite (HA) as carrier.
Modified ridge split with rhPDGF-BB hydrated crushed cortical freeze-dried bone allograft and collagen membrane using
Deproteinized bovine bone added to autologous bone vs. deproteinized bovine bone added to autologous bone with rhPDGF-BB.
rhPDGF-BB in conjunction with autogenous bone and anorganic bovine-derived bone mineral and a barrier membrane to reconstruct a severe alveolar posterior maxillary bone defect.
rhBMP-2
rhPDGF-BB in conjunction with an overlying titanium mesh in posterior mandibular ridge defect.
Autogenous bone in conjunction with rhPDGF-BB and barrier membrane.
1:1 ratio of autogenous bone graft and deproteinized bovide bone particles with rhPDGF-BB.
Xenogenic bone substitute and collagen membrane coated with rhBMP-2 and implant insertion.
